# Existence and Uniqueness of Solution for a Class of Stochastic Differential Equations

**DOI:** 10.1155/2013/132923

**Published:** 2013-09-19

**Authors:** Junfei Cao, Zaitang Huang, Caibin Zeng

**Affiliations:** ^1^Department of Mathematics, Guangdong University of Education, Guangzhou 510310, China; ^2^School of Mathematical Sciences, Guangxi Teachers Education University, Nanning 530023, China; ^3^Department of Mathematics, South China University of Technology, Guangzhou 510640, China

## Abstract

A class of stochastic differential equations given by *dx*(*t*) = *f*(*x*(*t*))*dt* + *g*(*x*(*t*))*dW*(*t*),  *x*(*t*_0_) = *x*_0_,  *t*_0_ ≤ *t* ≤ *T* < +∞, are investigated. Upon making some suitable assumptions, the existence and uniqueness of solution for the equations are obtained. Moreover, the existence and uniqueness of solution for stochastic Lorenz system, which is illustrated by example, are in good agreement with the theoretical analysis.

## 1. Introduction

Stochastic differential equations (SDEs) play an important role in characterizing many social, physical, biological, and engineering problems. They are important from the viewpoint of applications since they incorporate (natural) randomness into the mathematical description of the phenomena and provide a more accurate description of it. Therefore, the theory of SDEs has developed quickly, the investigation for SDEs has attracted considerable attention of researchers, and many qualitative theories of SDEs have been obtained (see [1–9] and the references therein).

The existence and uniqueness of solution are among the most basic and key topics in qualitative theory of SDEs. In the last two decades, the existence and uniqueness of solution for SDEs have been considered in many publications such as [10–14] and the references therein. Especially, Mao had investigated the stochastic differential equations:

(1) $$\begin{array}{c} dX\left(t\right)=f\left(X\left(t\right),t\right)dt+g\left(X\left(t\right),t\right)dB_{t},\;\;X\left(t_{0}\right)=X_{0}, \end{array}$$

on the closed interval [*t*_0_, *T*], *t*_0_ ≤ *T*, in his book [14], and obtained that if Lipschitz condition

(2) ||f(X,t)−f(X−,t)||2∨||g(X,t)−g(X−,t)||2 ≤K−||X−X−||2, X,X−∈ℝd,  t∈[t0,T],

and linear growth condition:

(3) ||f(X,t)||2∨||g(X,t)||2≤K(1+||X||2),          (X,t)∈ℝd×[t0,T],

hold, then (1) had a unique solution *X*(*t*) satisfying *X*(*t*) ∈ *ℳ* ^2^([*t*_0_, *T*],  ℝ^*d*^). Furthermore, Mao [14] also discussed stochastic functional differential equations with finite delay:

(4) dX(t)=f(Xt,t)dt+g(Xt,t)dBt,  Xt0=ξ, t0≤t≤T<+∞,

where *X*_*t*_ = {*X*(*t* + *θ*):−*τ* ≤ *θ* ≤ 0} could be considered as a *C*([*t*_0_, *T*],  ℝ^*d*^)-value stochastic process. The initial value *X*_*t*_0__ = *ξ* = {*ξ*(*θ*):−*τ* ≤ *θ* ≤ 0} is an *F*_*t*_0__-measurable *C*([*t*_0_, *T*],  ℝ^*d*^)-value random variable such that *𝔼*||*ξ*||^2^ < *∞*. For (4), if uniform Lipschitz condition

(5) ||f(φ,t)−f(ϕ,t)||2∨||g(φ,t)−g(ϕ,t)||2 ≤K−||φ−ϕ||2, K−>0,

for any *φ*, *ϕ* ∈ *C*([*t*_0_, *T*];  ℝ^*d*^), *t* ∈ [*t*_0_, *T*], and linear growth condition

(6) ||f(φ,t)||2∨||g(φ,t)||2≤K(1+||φ||2),      (φ,t)∈ℝd×[t0,T],  K>0,

are satisfied, then (4) had a unique solution *X*(*t*); moreover, *X*(*t*) ∈ *ℳ* ^2^([*t*_0_, *T*],  ℝ^*d*^).

We also mention that, following Mao [14], many papers were devoted to improving the results of Mao [14] by weakening the Lipschitz conditions on coefficients (e.g., see Jiang and Wang [15], Fei [16], Caraballo et al. [17], Wu et al. [18], Jiang and Shen [19], Taniguchi [20], Fan and Jiang [21], Bao and Hou [22], Ren et al. [23], Taniguchi [24], Wang and Huang [25], Lin [26], Xie [27], Fei [28], Ren and Zhang [29] and Zhang [30], etc.). In particular, Caraballo et al. [17], Taniguchi [20], Bao and Hou [22], and Taniguchi [24] have studied the existence and uniqueness of solutions to SDEs under the non-Lipschitzian condition.

To the best of our knowledge, most of the results on existence theory for SDEs focused on the case of the coefficient satisfying linear growth condition; however, the results on existence theory for SDEs without linear growth condition were discussed seldom. In this paper, without linear growth condition, some new criteria ensuring the existence and uniqueness of solutions for a class of SDEs are firstly established. These criteria improve, complement a number of existing results, and handle some cases not covered by known criteria. The results obtained in this paper not only improve the previous conclusion in the case of linear growth condition, but also can be applied in the existence and uniqueness of solution for the stochastic Lorenz system for weather forecasting, some other systems, and so on.

The rest of this paper is organized as follows. In Section 2, some relating notations and preliminary facts are introduced.  Section 3 obtains the existence and uniqueness of solution for a class of SDEs without linear growth condition. In Section 4, an interesting examples is given to show the effectiveness of our results.

## 2. Preliminaries

This section is concerned with some notations and preliminary results which are used in what follows.

In this paper, we adopt the symbols as follow: ℝ^*n*^ denotes the usual *n*-dimensional Euclidean space, ||·|| denotes norm in ℝ^*n*^. If *A* is a vector or a matrix, its transpose is denoted by *A* ^*T*^; if *A* is a matrix, its trace norm is represented by $||\cdot ||=\sqrt{\text{trace}(A^{T}A)}$. Let (*Ω*, *ℱ*, *ℙ*) be a complete probability space with a filtration {*ℱ*_*t*_}_*t*∈[*t*_0_,+*∞*)_ satisfying the usual conditions (i.e., it is increasing and right continuous). *ℱ*_*t*_0__ is independent of the *σ*-field generated by {*W*(*t*) − *W*(*t*_0_) : *t*_0_ ≤ *t* ≤ *T*} and contains all *ℙ*-null sets. *W*(*t*) is a given *m*-dimensional standard Brownian motion.

Consider *n*-dimensional stochastic differential equations:

(7) dx(t)=f(x(t))dt+g(x(t))dW(t),  x(t0)=x0, t0≤t≤T<+∞,

where *x*(*t*) = (*x*_1_(*t*), *x*_2_(*t*),…, *x*_*n*_(*t*))^*⊤*^, *x*_0_ = (*x*_1_(*t*_0_), *x*_2_(*t*_0_),…, *x*_*n*_(*t*_0_))^*⊤*^ is independent of *ℱ*_*t*_, and satisfies *𝔼*||*x*_0_||^2^ < +*∞*, *W*(*t*) is an *m*-dimensional Wiener process, *f*(*x*(*t*)) = (*f*_1_(*x*(*t*)), *f*_2_(*x*(*t*)),…*f*_*n*_(*x*(*t*)))^*⊤*^, *g*(*x*(*t*)) is a *n* × *m* matrix. Our purpose is to find the solution for (7). Hence, we will show the existence, uniqueness theorem and the properties of solution for (7) in the next section. Moreover, to illustrate the effectiveness of our results, we prove the existence and uniqueness of solution for the stochastic Lorenz system.

To guarantee the existence and uniqueness of solution for (7), the following conditions, instead of Lipschitz and linear growth conditions, are described.

(*H*_1_)  *f*(*x*(*t*)) satisfies the Lipschitz condition; moreover,

(8) $$\begin{array}{c} \langle f\left(x\right),x\rangle \leq k_{1}+k_{2}{||x||}^{2}; \end{array}$$

(*H*_2_)  *g*(*x*(*t*)) satisfies the Lipschitz condition and the linear growth condition:

(9) $$\begin{array}{c} {||g\left(x\right)||}^{2}\leq k^{2}\left(1+{||x||}^{2}\right), \end{array}$$

where 〈·, ·〉 denotes the inner product of ℝ^*n*^ and *k*_1_, *k*_2_, and *k* are constants.

## 3. The Existence and Uniqueness Theorem

In this section, we start to study the existence and uniqueness of solution for (7) without linear growth condition. To complete our main results, we need to prepare several lemmas which will be utilized in the sequel.

**Lemma 1**

Let (*H*_1_) and (*H*_2_) be satisfied. Setting *χ*_*N*_ ∈ *C* ^1^(ℝ^*n*^, ℝ) with

(10) χN(x)={1,for  ||x||≤N,0,for  ||x||≥N+1,

and *f*_*N*_(*x*) = *χ*_*N*_(*x*)*f*(*x*), and then the modified stochastic differential equations:

(11) $$\begin{array}{c} dx_{N}=f_{N}\left(x_{N}\right)dt+g\left(x_{N}\right)dW_{t},\\ x_{N}\left(t_{0}\right)=x_{0},\;t_{0}\leq t\leq T<+\infty , \end{array}$$

possesses a continuous almost sure unique *ℱ*_*t*_ measurable solution process.

**Proof**

As the truncation function *χ*_*N*_ ∈ *C* ^1^(ℝ^*n*^, ℝ), *f*_*N*_(*x*) remains differentiable and its derivative is both continuous and has a compact support. Hence *f*_*N*_(*x*) is bounded and satisfies linear growth condition as well as Lipschitz condition. According to (*H*_2_), *g*(*x*) satisfies linear growth condition and Lipschitz condition. Therefore, the assertion follows by the usual existence and uniqueness theorem (see Gihman and Skorohod [31], p. 37–47).

**Lemma 2 (see [32])**

Let *C*, *D* be both positive and semidefinite Hermite matrix, and *k* is a positive integer; then

(12) $$\begin{array}{c} \text{ trace }{\left(CD\right)}^{k}\leq {\left(\text{ trace }\left(C\right)\right)}^{k}{\left(\text{ trace }\left(D\right)\right)}^{k}. \end{array}$$

We compute the *p*th norm of the solution for (11) as follows. By Itô formula w.r.t. the function *h*(*x*) = ||*x*||^*p*^ = (∑_*i*=1_^*n*^ *x*_*i*_^2^)^*p*/2^ for *p* being even, one obtains

(13) d||xN||p=p||xN||p−2(f(xN), xN)dt+p(p2−1)||xN||p−4trace(xNxNTg(xN)  × gT(xN))dt+p2||xN||p−2 trace(g(xN)gT(xN))dt+p||xN||p−2xNTg(xN)dW(t).

According to Lemma 2, one has

(14) $$\begin{array}{c} \text{trace}\left(x_{N}^{T}x_{N}g^{T}\left(x_{N}\right)g\left(x_{N}\right)\right)\leq {||x_{N}||}^{2}{||g\left(x_{N}\right)||}^{2}. \end{array}$$

Together with (*H*_1_), the differential of the *p*th norm is given by

(15) d||xN||p=(pk1||xN||p−2+pk2||xN||p)dt+p(p−1)2||xN||p−2||g(xN)||2dt+p||xN||p−2xNTg(xN)dW(t)+ξ(t)dt,

where *ξ*(*t*) ≤ 0 is an adapted process that compensates all the estimation made and could be computed explicitly.

**Lemma 3**

Let *p* ∈ *ℕ* be even, and then there exists a constant *C*_*p*_ = *C*(*T*, *E*||*x*_0_||^*p*^, *p*) independent of *N* such that *E*||*x*_*N*_||^*p*^ < *C*_*p*_ for *t* ∈ [*t*_0_, *T*].

**Proof**

For *M* ∈ *ℕ*, define stopping time *τ*_*M*_ = inf⁡{*t* ∈ [*t*_0_, *T*] : ||*x*_*N*_(*t*)|| ≥ *M*}. Note that

(16) $$\begin{array}{c} \overset{t\wedge \tau_{M}}{\underset{t_{0}}{\int }}f\left(s\right)ds\leq \overset{t}{\underset{t_{0}}{\int }}f\left(s\wedge \tau_{M}\right)ds\;\text{for}\;f\left(t\right)\geq 0. \end{array}$$

Since ||*x*_*N*_(*t*∧*τ*_*M*_)||^*q*^ ≥ *M* ^*q*^ for all *q* > 0, using (*H*_2_) and the above inequality, one concludes that integrand of the stochastic integral is bounded; hence,

(17) $$\begin{array}{c} 𝔼\left(\overset{t\wedge \tau_{M}}{\underset{t_{0}}{\int }}p{||x_{N}\left(s\right)||}^{p-2}x_{N}{\left(s\right)}^{T}g\left(x_{N}\left(s\right)\right)dW\left(s\right)\right)=0. \end{array}$$

Form (*H*_2_), we get the following inequality:

(18) 𝔼||xN(t∧τM)||p≤𝔼||x(t0)||p+∫t0t[(pk1+k2p(p−1)2)   ×𝔼||xN(s∧τM)||p−2   +(pk2+k2p(p−1)2)   ×𝔼||xN(s∧τM)||p]ds.

Let *d*_1_ = *pk*_2_ + *k* ^2^ *p*(*p* − 1)/2, *d*_2_ = *pk*_1_ + *k* ^2^ *p*(*p* − 1)/2, then (18) becomes

(19) 𝔼||xN(t∧τM)||p ≤𝔼||x(t0)||p   +∫t0t(d1𝔼||xN(t∧τM)||p      + d2𝔼||xN(t∧τM)||p−2)ds,

which implies

(20) 𝔼||xN(t∧τM)||2 ≤𝔼||x(t0)||2  +∫t0t(d1𝔼||xN(s∧τM)||2+d2)ds.

Gronwall inequality implies that there exists a constant *C*_2_ such that

(21) $$\begin{array}{c} \underset{t\in \left[t_{0},T\right]}{\sup}𝔼{||x_{N}\left(s\wedge \tau_{M}\right)||}^{2}\leq C_{2}. \end{array}$$

By inductive method, there exist a constant *C*_*p*_ such that

(22) 𝔼||xN(s∧τM)||p≤𝔼||x(t0)||p+∫t0t(|d1|𝔼||xN(s∧τM)||p +|d2|Cp−2)ds≤Cp.

It remains to show that the stopping time satisfies *τ*_*M*_ → *T* for *M* → +*∞*. By the continuity of the solution *x*_*N*_(*t*) in *t*, the norm ||*x*_*N*_(*t*∧*τ*_*M*_)||^*p*^ is bounded; therefore, it converges *ω*-wise to ||*x*_*N*_(*t*)||^*p*^ as *M* → +*∞*. Since the norm is nonnegative and bounded for all *M* ∈ *ℕ*, for *t* ≤ *T*, Fatou Lemma implies that

(23) 𝔼||xN(t)||p≤𝔼(lim⁡M→+∞||xN(t∧τM)||p)≤liminf⁡M→+∞||xN(t∧τM)||p≤Cp.

**Lemma 4**

Let *p* ∈ *ℕ* be even, and then there exists a constant ${\tilde{C}}_{p}=\tilde{C}(T,E{||x_{0}||}^{p},p)$ independent of *N* such that $𝔼\underset{}{\sup_{t\in [t_{0},T]}}{||x_{N}(t)||}^{p}<{\tilde{C}}_{p}$ for *t* ∈ [*t*_0_, *T*].

**Proof**

From (15), it follows that

(24) ||xN(t∧τM)||2=||x(t0)||2+∫t0t∧τM2k2||xN(s)||2ds+∫t0t∧τM(2k1+||g(xN(s))||2)ds+∫t0t∧τM2xNT(s)g(xN(s))dW(s)+∫t0t∧τMξ(s)ds,

which implies

(25) ||xN(t∧τM)||2≤||x(t0)||2+∫t0t∧τM2|k2|||xN(s)||2ds+∫t0t∧τM(2|k1|+||g(xN(s))||2)ds+∫t0t∧τM2xNT(s)g(xN(s))dW(s).

Taking to the power *p*/2 to obtain an expression for ||*x*_*N*_(*t*∧*τ*_*M*_)||^*p*^, one has

(26) ||xN(t∧τM)||p≤|||x(t0)||2+∫0t∧τM2|k2|||xN(s)||2ds‍ +∫t0t∧τM(2|k1|+||g(xN(s))||2)ds +∫t0t∧τM2xNT(s)g(xN(s))dW(s)|p/2.

According to |∑_*i*=1_^*k*^ *a*_*i*_|^*m*^ ≤ *k* ^*m*−1^∑_*i*=1_^*k*^|*a*_*i*_|^*m*^ for *m* ≥ 1, one obtains

(27) 𝔼sup⁡t∈[t0,T]||xN(t∧τM)||p ≤4(p−2)/2𝔼||x(t0)||p  + 4(p−2)/2𝔼|∫t0T2|k2|||xN(s)||2ds|p/2  +4(p−2)/2𝔼|∫t0T(2|k1|+||g(xN(s))||2)ds|p/2  +4(p−2)/2𝔼sup⁡t∈[t0,T]|∫t0t∧τM2xNT(s)g(xN(s))dW(s)|p/2 =4(p−2)/2E||x(t0)||p+U1+U2+U3.

Since the solution *x*_*N*_(*t*) for (11) is *ℱ*_*t*_ measurable for *t* ≥ *t*_0_ and continuous in *t*, the norm ||*x*_*N*_(*t*)|| is *ℱ*_*t*_ ⊗ *ℬ*([*t*_0_, *t*])-measurable (see Wentzell [33], p. 89, p. 18). Therefore, applying (*H*_2_), together with Hölder inequality to remove the powers outside the deterministic integrals *U*_1_ and *U*_2_, changing expectation and integration by Fubini theorem and using Lemma 3, one obtains

(28) U1=4(p−2)/2𝔼|∫t0T2|k2|||xN(s)||2ds|p/2≤C~p1,U2=4(p−2)/2𝔼|∫t0T(2|k1|+||g(xN(s))||2)ds|p/2≤4(p−2)/2T(p−2)/2 ×∫t0T(2|k1|p/2+kp+kpC2)ds,

which is bounded by a constant ${\tilde{C}}_{p}^{2}$. To further estimate the stochastic integral *U*_3_, using the Burkholder-Davis-Gundy (see Mao [14], p. 7), one obtains

(29) U3=𝔼sup⁡t∈[t0,T]|∫t0txNT(s)g(xN(s))dW(s)|p/2≤kp𝔼|∫t0T||xN(s)g(xN(s))||2|p/4ds,

with the constant

(30) kp={(64p)p/4,for  0<p<4,(pp/2+12p/2+2(p/2−1)p/2−1)p/4,for  p≥4.

For *p* = 2, using $\sqrt{x}\leq 1+x$ and, for *p* > 2, Hölder inequality to remove the powers outside the integral on the right side of (29), afterwards, one proceeds similar to handle the Lebesgue integrals *U*_1_ and *U*_2_, which leads to a constant ${\tilde{C}}_{p}^{3}$. Combining the above discussion, one obtains

(31) 𝔼sup⁡t∈[t0,T]||xN(t∧τM)||p≤4(p−2)/2𝔼||x0||p+C~p1+C~p2+C~p3≤C~p.

To complete the proof one needs to show *τ*_*M*_ → *T* for *M* → +*∞*. Mentioning that the solution *x*_*N*_(*t*) is continuous in *t*, thus *𝔼* sup⁡_*t*∈[*t*_0_,*T*]_||*x*_*N*_(*t*∧*τ*_*M*_)||^*p*^ is bounded and converges *ω*-wise, for *M* → +*∞*, to *𝔼* sup⁡_*t*∈[*t*_0_,*T*]_||*x*_*N*_(*t*)||^*p*^. Moreover, Fatou Lemma and (31) imply

(32) 𝔼sup⁡t∈[t0,T]||xN(t)||p=𝔼lim⁡M→+∞sup⁡t∈[t0,T]||xN(t∧τM)||p≤liminf⁡M→+∞ 𝔼sup⁡t∈[t0,T]||xN(t∧τM)||p≤C~p.

Now, we state our first main result.

**Theorem 5**

Let (*H*_1_) and (*H*_2_) be satisfied, and then (7) possesses a unique almost sure continuous solution process.

**Proof**

It is easy to see that the uniqueness follows from the Lipschitz condition fulfilled by the coefficients (see Remark 2 in Gihman and Skorohod [31], p. 45).

In the following, we only need to prove the existence of solution for (7).

Note that Lemma 1 ensures the existence of a solution *x*_*N*_(*t*) for (11). We firstly show that *x*_*N*_(*t*) converges to a function *x* ^0^(*t*) for *N* → +*∞*.

Again let *τ*_*N*_ denote the stopping time *τ*_*N*_ = inf⁡{*t* ∈ [*t*_0_, *T*] : ||*x*_*N*_(*t*)|| ≥ *N*} for *N* ∈ *ℕ*. From Chebyshev inequality and Lemma 4, it follows that

(33) ℙ{τN<T}≤ℙ{sup⁡t∈[t0,T]||xN(t)||p≥N}≤𝔼(sup⁡t∈[t0,T]⁡||xN(t)||p)NP≤C~(T,E||x0||p,p)NP→0,as  N→+∞.

Note that this states slightly more than convergence in probability of *τ*_*N*_ → *T*. One can find, for almost every *ω* ∈ *Ω*, an *N*_0_(*ω*) such that *τ*_*N*_0_(*ω*)_ = *T*. Moreover, one has *τ*_*N*′_ ≥ *τ*_*N*_ and *x*_*N*′_^*x*_0_^(·, *ω*) = *x*_*N*_^*x*_0_^(·, *ω*) (almost sure) on [*t*_0_, *τ*_*N*_] for all *N*′ ≥ *N* (see Gihman and Skorohod [14], p. 4). Thus, if *τ*_*N*_ = *T*, then *τ*_*N*′_ = *T* for all *N*′ ≥ *N*. From the above discussion, it follows that the set {*ω* : *τ*_*N*_(*ω*) = *T*} is monotonously increasing and converges to *Ω*, for *N* → +*∞*. We point out once more that if *τ*_*N*_0_(*ω*)_ = *T*, then one can express, for almost *ω* ∈ *Ω*, the limit function by *x*(·, *ω*) = *x*_*N*′_(·, *ω*) for all *N*′ ≥ *N*_0_(*ω*). (Actually *x*_*N*_^*x*_0_^(·) is only a version of *x*(·) on [*t*_0_, *τ*_*N*_]; i.e., there exists an exceptional *ℙ*-null set *𝒩*(*N*). Note that there are countable many such null sets, so that the union over all the *ℙ*-null set is again a null set.) Therefore, *x*_*N*_(*t*) converges uniformly in *t* to *x* ^0^(*t*), together with *x*_*N*_(*t*) is continuous in *t*, *x* ^0^(*t*) is further continuous in *t*.

Secondly, we further show that the limit function *x* ^0^(*t*) is a real solution for (7).

For *t* = *t*_0_, this is true because *x*_*N*_(*t*_0_) = *x*_0_ for all *N* ∈ *ℕ*. For *t* ∈ (*t*_0_, *T*], one considers the limit function of *x*_*N*_(*t*) for *N* → +*∞*. According to *f*_*N*_(*x*_*N*_(*t*∧*τ*_*N*_)) = *f*(*x*(*t*∧*τ*_*N*_)) and *x*_*N*_(*t*∧*τ*_*N*_) = *x*(*t*∧*τ*_*N*_) for *t* ≤ *T*, the almost sure convergence of *τ*_*N*_ to *T* implies

(34) ℙ{sup⁡t∈[t0,T]||∫t0t(f(xN(s))−f(x(s)))ds+∫t0t(g(xN(s))−g(x(s)))dW(t)||>0} ≤ℙ{τN<t}→0, as  N→+∞.

Therefore, *x* ^0^(·) is a solution for (7) on [*t*_0_, *T*]. The proof is completed.

Under the assumptions (*H*_1_) and (*H*_2_), the solution has some important properties.

**Corollary 6**

The solution *x*(*t*) for (7) has the following properties.

(i) *x*(*t*) is a *ℱ*_*t*_ × *ℬ*([*t*_0_, *T*])-measurable homogeneous Markov process.
(ii) Let *𝔼*||*x*_0_||^*p*^ < +*∞* for a fixed even *p*, and then there exists a constant ${\tilde{C}}_{p}=\tilde{C}(T,E{||x_{0}||}^{p},p)$ such that
  (35) $$\begin{array}{c} 𝔼\underset{t\in \left[t_{0},T\right]}{\sup}{||x\left(t\right)||}^{p}\leq {||x\left(t\right)||}^{p}\leq {\tilde{C}}_{p},\;\forall t\in \left[t_{0},T\right]. \end{array}$$

Furthermore, for every deterministic and bounded set *B* ⊂ ℝ^*n*^, the constant $\sup_{x_{0}\in B}{\tilde{C}}_{p}$ is finite (*x*_0_ is deterministic).

**Proof**

(i) Because the coefficients *f*_*N*_(*x*_*N*_), *g*(*x*_*N*_) of (11) are independent of *ω*, *t* respectively, and fulfill both linear growth condition and Lipschitz condition, the solution *x*_*N*_(*t*) is a homogeneous Markov process by Theorem 1 in Gihman and Skorohod [31] (Section 10). Therefore, the solution *x*(*t*) of system (7) is also a homogeneous Markov process since *x*_*N*_(*t*) uniformly converges to *x*(*t*).

(ii) From the proof of Lemma 4, it is to see that there exists the constant ${\tilde{C}}_{p}$ in (ii). Note that *𝔼*||*x*(*t*)||, *t* ∈ [0, *T*], can be bounded by a probably more accurate constant using Lemma 3. Every bounded set *B* is contained in a ball of appropriate radius *R* and center zero. Setting ||*x*_0_|| = *R*, the assertion follows from the linear dependence of the bounding constant (cf. (23) and (31) resp.).

**Corollary 7**

Let 2*k*_2_ + *k* ^2^ < 0, and then there exists a constant *C*(*𝔼*||*x*_0_||^2^) such that

(36) $$\begin{array}{c} \underset{t\in \left(t_{0},+\infty \right)}{\sup}E{||x\left(t\right)||}^{2}<C\left(E{||x_{0}||}^{2}\right). \end{array}$$

**Proof**

The assumptions imply that the constant *d*_1_ in the proof of Lemma 3 is negative. Hence, there exist a bounded constant only depending on the initial condition, but not on *t*.

## 4. Applications

In this section, we prove the existence and uniqueness of solution for the stochastic Lorenz system for weather forecasting as an application to illustrate the effectiveness of our results.

The Lorenz system was introduced by Lorenz [34]. For the physical meaning of the system, the reader is referred to Peitgen et al. [35]. Forces not described by those equations are assumed to be random. We model these influences by white noise. This leads to the stochastic Lorenz system which has been described in detail in Arnold [36] and has been intensively studied in [6, 37] and so forth.

**Definition 8**

Let *x* = (*x*, *y*, *z*)^*⊤*^ ∈ ℝ^3^, *β*, *r*, *σ* > 0 be constants, then the stochastic Lorenz system is defined by

(37) $$\begin{array}{c} dx=\left(-Ax+f\left(x\right)\right)dt+g\left(x\right)dW\left(t\right),\;\;x\left(t_{0}\right)=x_{0}, \end{array}$$

the two parts of the drift are given by

(38) $$\begin{array}{c} A=\left(\begin{array}{ccc} \sigma & -\sigma & 0\\ -r & 1 & 0\\ 0 & 0 & \beta \end{array}\right),\;\;f\left(x\right)=\left(\begin{array}{c} 0\\ -xz\\ xy \end{array}\right), \end{array}$$

and the noise term *g*(*x*) : ℝ^3^ → (3 × *m*) matrix satisfies Lipschitz as well as linear growth condition.

**Remark 9**

If one considers the noise to act in Stratonovich form, one talks about the Stratonovich Lorenz system. Note that the Stratonovich and the corresponding Itô Lorenz system are equivalent up to an additional drift term (see Arnold [38], p. 181).

**Theorem 10**

The stochastic Lorenz system (37) exists a unique solution.

**Proof**

Let *x*′ = *x*, *y*′ = *y*, *z*′ = *z* − *r* − *σ*, and *ω* = (*x*′, *y*′, *z*′)^*⊤*^ ∈ ℝ^3^, and one has

(39) $$\begin{array}{c} d\omega =\left(-D\omega -B+\overset{-}{f}\left(\omega \right)\right)dt+\overset{-}{g}\left(\omega \right)dW\left(t\right). \end{array}$$

The three parts of the drift are given by

(40) $$\begin{array}{c} D=\left(\begin{array}{ccc} \sigma & -\sigma & 0\\ \sigma & 1 & 0\\ 0 & 0 & \beta \end{array}\right),\;\;\overset{-}{f}\left(\omega \right)=\left(\begin{array}{c} 0\\ -x^{\prime }z^{\prime }\\ x^{\prime }y^{\prime } \end{array}\right),\\ B=\left(\begin{array}{c} 0\\ 0\\ \beta \left(r+\sigma \right) \end{array}\right). \end{array}$$

Obviously, the noise term $\overset{-}{g}(\omega ):{\mathbb{R}}^{3}\to (3\times m)$ matrix remains satisfying Lipschitz as well as linear growth condition. According to (40), one has $\langle \overset{-}{f}(\omega ),\omega \rangle =0$. Moreover,

(41) 〈−Dω−B,ω〉=−σx′2−y′2−βz′2−β(r+σ)z′≤(−a+12)||ω||2+β2(r+σ)22,

where *a* = min⁡{*σ*, 1, *β*}. Hence according to Theorem 10, system (39) exists a unique solution, so is system (37).

## 5. Conclusion

This paper considers the problems of existence and uniqueness of solution for a class of stochastic differential equations whose nonlinear part does not satisfy linear growth condition. Some new criteria ensuring the existence and uniqueness of solution were presented. These criteria extend, improve, complement a number of results about the existence and uniqueness of solution, and handle some cases not covered by known criteria. Furthermore, these criteria are important in applications such as stochastic Lorenz system for weather forecasting, some other systems, and so on.
